# Effectiveness of Platform-Based Robot-Assisted Rehabilitation for Musculoskeletal or Neurologic Injuries: A Systematic Review

**DOI:** 10.3390/bioengineering9040129

**Published:** 2022-03-22

**Authors:** Anil Babu Payedimarri, Matteo Ratti, Riccardo Rescinito, Kris Vanhaecht, Massimiliano Panella

**Affiliations:** 1 Department of Translational Medicine (DIMET), Università del Piemonte Orientale, 28100 Novara, Italy; matteo.ratti@uniupo.it (M.R.); 10033325@studenti.uniupo.it (R.R.); massimiliano.panella@med.uniupo.it (M.P.); 2 Department of Public Health and Primary Care, Leuven Institute for Healthcare Policy, KU Leuven, 3000 Leuven, Belgium; kris.vanhaecht@kuleuven.be; 3 Department of Quality Management, University Hospitals Leuven, University of Leuven, 3000 Leuven, Belgium

**Keywords:** robot-assisted training, neurorehabilitation, orthopedic rehabilitation, clinical effectiveness, platform-based robotic rehabilitation, Parkinson’s disease, stroke, spinal cord injuries

## Abstract

During the last ten years the use of robotic-assisted rehabilitation has increased significantly. Compared with traditional care, robotic rehabilitation has several potential advantages. Platform-based robotic rehabilitation can help patients recover from musculoskeletal and neurological conditions. Evidence on how platform-based robotic technologies can positively impact on disability recovery is still lacking, and it is unclear which intervention is most effective in individual cases. This systematic review aims to evaluate the effectiveness of platform-based robotic rehabilitation for individuals with musculoskeletal or neurological injuries. Thirty-eight studies met the inclusion criteria and evaluated the efficacy of platform-based rehabilitation robots. Our findings showed that rehabilitation with platform-based robots produced some encouraging results. Among the platform-based robots studied, the VR-based Rutgers Ankle and the Hunova were found to be the most effective robots for the rehabilitation of patients with neurological conditions (stroke, spinal cord injury, Parkinson’s disease) and various musculoskeletal ankle injuries. Our results were drawn mainly from studies with low-level evidence, and we think that our conclusions should be taken with caution to some extent and that further studies are needed to better evaluate the effectiveness of platform-based robotic rehabilitation devices.

## 1. Introduction

In the last several years, the use of robotic-assisted rehabilitation has increased significantly [1,2]. Compared with traditional care, robotic rehabilitation can be better performed at high intensity and frequency, and can continuously monitor exercise performance so that the level of treatment can be better adapted to the patient’s needs [3], and can generate more appropriate movements and forces during training [4,5].

Two major types of robotic rehabilitation devices are available. The first one consists of wearable devices: robotic orthoses [6] and exoskeletons [7] for correcting the gait pattern of patients and improving ankle performance during walking. The second one includes platform-based devices that are designed solely to improve ankle performance [8,9]. These technologies have a fixed platform and a movable footplate that can be used with a single degree of freedom (DOF) [10] or multiple degrees of freedom (DOFs) [11,12]. Platform-based robotic rehabilitation allows for complex and specialized spatial movements [11,12,13,14]. Their architectures provide the device with high stiffness, balanced force distribution, and improved adaptability to the mechanical properties of human ankle joints [13]. The utilization of platform-based robotic rehabilitation can help the patients recovering from neurological conditions (e.g., stroke, brain injury, spinal cord injury, and cerebral palsy) and musculoskeletal disorders (e.g., post-traumatic lower-limb disorders).

Recently, systematic reviews (SRs) have examined the effectiveness of wearable robotic technologies: exoskeletons and orthoses for upper limbs [15]; exoskeletons for lower limbs in neuromuscular impairments [16]; and individuals with cerebral palsy [17]. However, evidence on how platform-based robotic technologies can positively impact disability recovery is still lacking, and it is unclear which intervention is most effective in individual cases. Therefore, we performed this systematic review with the main aim of first providing a comprehensive evaluation of the evidence-based effectiveness of platform-based rehabilitation robotics for people with musculoskeletal or neurological injuries. Secondly, we aim to show the current state of platform-based rehabilitation robotics technology. Finally, these results should enable physiotherapists to safely use platform-based robots in rehabilitation settings.

## 2. Materials and Methods

This systematic review was conducted according to the Preferred Reporting Items for Systematic Reviews and Meta-Analyses (PRISMA) checklist [18].

### 2.1. Search Strategy

A literature search was performed in the following electronic databases: PubMed, Excerpta Medical Database (EMBASE), Cochrane Database of Systematic Reviews (CDSR), and Scopus. The medical subject headings (MeSH) used were ”robotics” and ”rehabilitation”, “robotics” and “rehabilitate*” or “treat*” and “effectiveness of robotics.” In addition, we performed a free search on Google Scholar by using keywords (such as ”platform-based robotics” and “robotic rehabilitation effectiveness”) and reviewed key references in relevant publications to make our search as systematic and complete as possible. We also used a snowball search strategy, which means the reference lists of selected and published systematic review articles were manually searched to identify other possible eligible studies. The snowball search was limited to articles published between January 2016 and August 2021. The Movendo technology website was also searched for the clinical trials [19].

### 2.2. Inclusion and Exclusion Criteria

We included all the studies that examined the clinical outcomes of platform-based robotic rehabilitation training. Both male and female participants (all ages) were included to allow for the generalization of the results. We considered studies with at least one ill subject in the intervention group rehabilitated with a platform-based robot and articles in English and Italian published (as of August 2021) in peer-reviewed journals or as conference papers (national and international), abstracts, or as gray literature. Studies that included only healthy subjects and/or evaluated outcomes with another type of robotic rehabilitation technology were excluded.

### 2.3. Selection Criteria

Based on the titles, a screening of the articles was performed by two independent reviewers (M.R. and R.R.) to identify relevant studies. One reviewer (M.R.) evaluated all article abstracts for eligibility after the screening of titles was completed. Abstracts that met the inclusion criteria were included in the full review. Otherwise, they were excluded. Studies which one reviewer (M.R.) was unsure whether to include were discussed by two reviewers (M.R., R.R.). Disagreements between the two reviewers were resolved by the inclusion of the third reviewer (A.B.P.) and discussion between these three reviewers. The full texts of the included articles were examined for the typology of platform-based technological rehabilitation robot studied, and the duration of training in the intervention group.

### 2.4. Assessment of Methodological Quality

The quality of all included studies was evaluated by two independent reviewers (M.R., A.B.P.) by using the Joanna Briggs Institute (JBI) critical appraisal tool [20]. The JBI critical appraisal tool is used to assess the methodological quality of studies. It consists of items (13 items for RCT, 11 for cohort studies, 8 for case studies) that address the internal validity and risk of bias of the studies, particularly confounding, selection, and information bias, as well as the importance of clear reporting. Disagreements between the two reviewers were resolved through the involvement of a third reviewer (K.V.) and discussions among the three reviewers. A high risk of bias was determined when positive responses were ≤49%; moderate risk of bias was assumed when risk of bias was between 50% and 69%; a low risk of bias was determined when positive responses were greater than 70%.

### 2.5. Data Extraction

A data extraction sheet was developed to collect data of interest. Three reviewers (M.P., K.V., and A.B.P) read all included articles. One author (A.B.P.) extracted the data from the articles, and later they were double-checked by two other authors (K.V., M.P.). The following information was extracted from each study: title, study design, clinical area, patient characteristics (type of disorder, number of subjects, gender, age), intervention group, outcome measures, and study conclusions. A qualitative synthesis was conducted for the included studies.

## 3. Results

### 3.1. Study Selection

**Figure 1:**
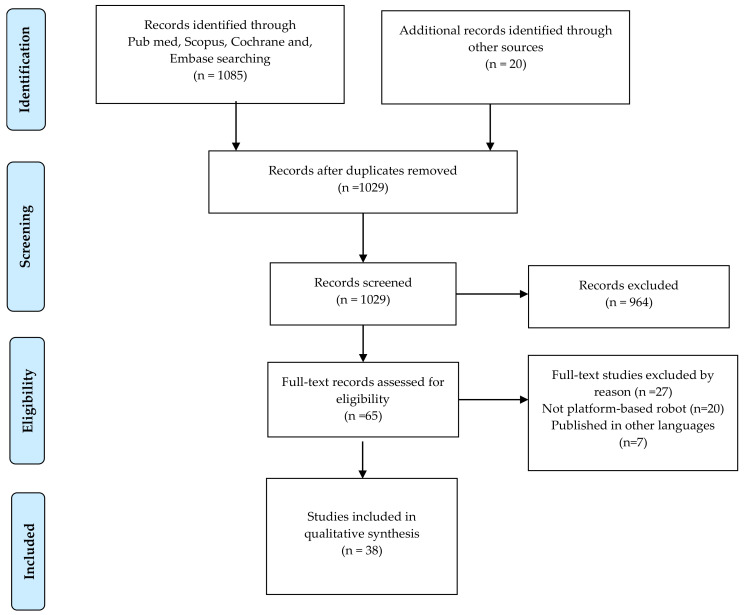
PRISMA Flow diagram of the records selection process.

We found 1085 entries in Medline (PubMed), Scopus, Cochrane Library, and EMBASE, and 20 additional entries came from Google Scholar and the Movendo technology website [21]. After removing 76 duplicate records, 1029 articles were selected for screening. After reading the title and abstract, 964 records were excluded because they were not relevant to our inclusion criteria. We then screened 65 full-text articles for eligibility. After reading the full text, 27 articles were excluded. Therefore, 38 studies were included in our review. The overall selection process is shown in Figure 1. The studies were conducted to develop, validate, and evaluate the effectiveness of platform-based rehabilitation robots. All the studies met the JBI criteria for the critical appraisal (Appendices 1, 2, and 3) [20]. A summary of quality assessments results is also reported in the Supplementary Materials (Tables S1 and S2). In brief, 25 studies had a low risk of bias with a high percentage of positive responses to the JBI tool questions, 10 studies were classified as having a moderate risk of bias, and the remaining three studies were classified as having a high risk of bias.

### 3.2. Study Characteristics

Table 1 describes the characteristics of the included studies. The study design included six RCTs with 156 participants, two prospective longitudinal studies, four clinical practice studies, and 26 case studies. Most participants suffered from ankle disabilities, mainly due to musculoskeletal injuries and strokes. In addition, traumatic or nontraumatic brain and spinal cord injuries as well as Parkinson’s disease, knee arthroplasty, and surgical reconstruction of the anterior cruciate ligament (ACL) also played a role in ankle disabilities.

Regarding the outcomes, as they are shown in Table 1, 12 studies investigated ankle-joint performance (e.g., ankle strength, ankle range of motion (ROM), and ankle motor control) [10,22,23,24,25,26,27,28,29,30,31,32]. Nineteen studies examined one or more of the following outcomes: components or parameters of the ankle ROM, trunk parameters (e.g., trunk control, trunk compensations, trunk variability, trunk acceleration, trunk stability), monopodal and bipodal balance, squat, and proprioceptive abilities [33,34,35,36,37,38,39,40,41,42,43,44,45,46,47,48,49,50,51]. Two studies examined gait function outcomes [52,53]. Four studies evaluated both ankle performance and gait function to validate the effect of robotic ankle rehabilitation devices [54,55,56,57]. One study [24] also evaluated the device performance by measuring pressure distribution on the footplate. Two studies [26,55] also used participant satisfaction as the main outcome. One study examined neuropsychological assessment with an emphasis on body representation (BR) and cognitive and linguistic functions [58].

### 3.3. Effectiveness of Platform-Based Rehabilitation Robots

We found the following types of platform-based robotic rehabilitation systems, and their effectiveness is described as follows.

#### 3.3.1. Rutgers Ankle Rehabilitation System

This robotic system has multiple DOF. It operates based on a parallel mechanism (PM) driven by linear actuators, and it is used specifically for ankle rehabilitation in cases of limited mobility [26]. Ten studies have been conducted using the Rutgers Ankle Rehabilitation System (Stewart platform type).

The Virtual Reality System (VR) Rutgers Ankle robot has been investigated in five studies [25,30,52,53,56] and the VR-based telerehabilitation system in one study [29] to rehabilitate individuals after stroke and measure its effects by using various criteria (training focused on gait and climbing speed, ankle and foot mobility, force production, coordination, ability to walk and climb stairs, velocity and distance walked, ankle mechanical strength, and the number of repetitions). The results showed improvements in gait and elevation speed (from 0% to 44% and 3% to 33%, respectively) [53], ankle motion accuracy, exercise duration and training efficiency, ankle mechanical strength, and the number of repetitions [29]. It has been also shown that rehabilitation can help to improve gait and climbing speed [30,53] and ankle muscle strength [25]. However, a robotic virtual reality system produced better results than a robot alone, although the benefits were minor in the gait of post-stroke patients [52].

Two studies investigated the effectiveness of the Rutgers ankle robot in patients with cerebral palsy (CP) [23,57]. Both studies found improvements in ankle strength, motor control, gain function, and patient quality of life [23,57].

Two studies evaluated the effectiveness of the Rutgers ankle haptic interface in patients with musculoskeletal ankle injuries [26,27]. One study showed improvements in range of motion (ROM), ability to generate torque, and mechanical work of the ankle [27]. Another study concluded that the robot can be used for the rehabilitation of patients with hyper-and hypomobile ankles [26].

#### 3.3.2. Ankle Stretching Robotic Rehabilitation System

This type of robotic rehabilitation system usually consists of a single DOF and is usually driven by a rotating motor for a specific application (e.g., ankle stretching). Robotic ankle-stretching devices have been used for rehabilitation in four studies.

Two studies [10,55] investigated an intelligent robotic stretching device in patients with ankle contracture and/or spasticity. Results showed that the treatment was more effective than existing approaches in terms of active and passive ROM, joint stiffness, viscous damping, and reflex excitability [10]. Improvements were noted in muscle strength, walking speed, and subjective sensation [55].

Robotic passive stretching and active training (with biofeedback via motivational games) for ankle rehabilitation has been studied in post-stroke individuals [28] and lower extremity (LE) impairments in children with CP [22]. Results showed improvements in the Modified Ashworth Scale (MAS), the Stroke Rehabilitation Assessment of Movement (STREAM), active dorsiflexion range, dorsiflexor muscle strength, and the Berg Balance Scale (BBS) [28], joint biomechanical properties, motor control performance, and functional capacity in balance and movement [22].

#### 3.3.3. Other Typology of Ankle Robotic Rehabilitation Devices

Homma et al. developed a training robot for ankle dorsiflexion/plantarflexion with a passive mechanical joint. Its effectiveness was evaluated based on pressure distribution on the footplate in a hemiplegic patient. It was unclear in the study whether pressure distribution could be used as an indicator of recovery. Therefore, the study concluded that its relationship to biological data should be further investigated [24].

Cordo et al. tested the efficacy of Assisted Movement with Enhanced Sensation (AMES) by using a robotic ankle device as a treatment for hemiplegia by using strength and joint alignment tests and motor function in post-stroke patients. Results showed improvements in most functional tests within six months [54].

Zhou et al. validated the treatment strategy of proprioceptive neuromuscular facilitation (PNF) with a robotic ankle–foot system in post-stroke patients over six weeks. Results showed improvements in ankle spasticity and/or contracture at the end of the study [32].

#### 3.3.4. ARBOT Rehabilitation Robot

ARBOT is a robotic prototype for ankle rehabilitation. The effectiveness of ARBOT was evaluated on 32 subjects (with ankle and/or hindfoot fractures) comparing conventional and ARBOT-assisted ankle rehabilitation. After a 4-week rehabilitation program, the experimental group showed a significant improvement in the proprioceptive test compared to the control group [31].

#### 3.3.5. Hunova Robotic Rehabilitation System

Hunova is a “platform-based” end-effector robot consisting of two electromechanical platforms with 2 DOF: one is located under the feet and the other under the seat. It is possible to perform exercises both sitting and standing. It allows passive (mobilization), active (with elastic or fluid resistance), proprioceptive, and assistive therapy (i.e., the device intervenes to complete the exercise when the patient needs it). The device can work in both static and dynamic modes. It is used for rehabilitation and evaluation of the sensory-motor function of the lower limbs and torso as well as balance [12]. Twenty studies have been conducted by using the Hunova robot in geriatrics, neurology, and orthopedics. Of these, ten were case studies, four were RCTs, two were prospective longitudinal studies, and four were clinical practice studies.

- Geriatric rehabilitation

Two studies focused on the assessment of balance parameters with Hunova in 96 elderly subjects [33,34]. One study showed that multifactorial fall risk assessment (including clinical and robotic variables) significantly improved the accuracy of fall-risk prediction [34]. Another study showed that balance parameters assessed with this robot were significantly correlated with functional performance in the Short Physical Performance Battery (SPPB) [33].

ii. Neurorehabilitation

Six studies investigated the effectiveness of robotic rehabilitation (training focused on balance and core stability, ankle mobility) in patients with Parkinson’s disease [35,36,37], stroke [39], cavernous malformation and hypertrophic olivary degeneration [44], and severe acquired brain injury [45]. One study focused on the robotic assessment of balance in sitting and standing in patients with Parkinson’s disease [38]. A study investigated multidisciplinary treatment (motor rehabilitation training, traditional physiotherapy and robotic rehabilitation, and psychological counselling) in a patient with somatoparaphrenia (SP) and misoplegia [58]. The results showed improvement in the Timed Up and Go (TUG) test, greater mobility and stability of the pelvis, load management in a seated position [35], dynamic balance, and walking speed [37,44], gait speed and ankle range of motion [45], reactive balance and postural control in an unstable condition, and proprioceptive control in standing and sitting [39]. However, in an RCT (treatment of balance with a robotic platform compared to a conventional rehabilitation), both types of training were found to improve balance, walking, and quality of life [36]. Although differences were noted in postural control, greater trunk sway was noted at the end of the study in association with a greater increase in center of pressure (CoP) backward displacement [38]. Maggio et al. have shown in a case study that an integrated psychological and motor approach can effectively rehabilitate patients with SP, even in the presence of misoplegia [58].

Four studies investigated the effectiveness of robotic assessment and training (focusing on balance, trunk control, dual-motor task with upper limb movements, strengthening, core stability) in individuals with spinal cord injuries (SCI) [40,41,42] and in a patient with retro-ocular frontal aneurysm with hemorrhage [43]. The results showed improvements in trunk control [41,42], dynamic balance and walking speed [43], as well as differences in motor performance at the end of the study [40].

iii. Orthopedic rehabilitation

Three studies investigated Hunova as an aid in rehabilitation, in patients with knee arthroplasty [48], post-traumatic lower limb disorders (to restore motor control and gait performance) [46], and ankle fracture-dislocation [49]. Three studies were performed for evaluation and rehabilitation training in patients after surgical reconstruction of the anterior cruciate ligament (ACL) [47] in a soccer player [50] and in a basketball player with chronic ankle instability [51].

The main results showed improvements in orthostatic stability, postural passages (such as sitting and standing in reactive balance) [48], left ankle performance (in terms of ROM and isometric and isokinetic strength) [49], proprioceptive tests [46], stability index (in dynamic balance tests), joint mobilization, muscle work, and proprioceptive recovery [47]. In two studies, the total performance index improved from 61% to 76% [50] and from 57% to 74%. The two functional areas trained (core and range of motion) [50,51], and the Foot and Ankle Ability Measure (FAAM) score also improved after rehabilitation [51].

## 4. Discussion

The purpose of our systematic review was to determine the evidence-based effectiveness of platform-based rehabilitation robots for patients with musculoskeletal or neurological disabilities. As a major finding, our study showed that patients with neurological impairments and musculoskeletal injuries can be effectively treated with platform-based robotic rehabilitation devices after their health status is stabilized. With the higher repetitions that robotic devices provide, patients can exercise more, which stimulates neural plasticity in neurological patients in the early stages of their recovery. Once patients can walk better, they can transition to conventional walking to further practice walking on different terrain, improve balance, and correct abnormal gait patterns.

Therefore, we think that platform-based robotic training should be routinely adopted in rehab clinics next to traditional physical therapy. In fact, physiotherapists can use robotic equipment as a multiplier to train more patients. Instead of traditional one-to-one practice, therapists can use robotic devices to treat more patients at the same time. This frees up valuable time for therapists to either train severe patients individually or practice more function-based tasks that require the integration of multiple motor skills. By spending their time on this higher value training activities and letting the robotic devices take over the “heavy” routine tasks, therapists can provide an appropriate level of personalized treatment to their patients and increase their efficiency.

Even though in our study we identified seven types of platform-based robotic rehabilitation systems and reported on their effectiveness [10,12,24,26,31,32,54], we could only draw conclusions for the VR-based Rutgers ankle and Hunova because of the availability of specific literature. In detail, we found that the VR-based Rutgers ankle and Hunova seemed to be the most effective robots for rehabilitation. Ankle rehabilitation using a VR-based Rutgers ankle robot has been shown to be effective in rehabilitating patients after stroke and various musculoskeletal ankle injuries [25,29,30,52,53,56]. We also found that rehabilitation treatment with Hunova is an innovative therapeutic option that can be combined with traditional rehabilitation in patients with Parkinson’s disease [35,36,37], a promising tool for the rehabilitation of stroke patients [39]. For spinal cord injuries (SCI), it can be a useful rehabilitation tool for assessment and training [40,41,42]. In addition, rehabilitation with the Hunova allows measurement of important parameters of static and dynamic stability and can focus on a complex sequence of exercises to restore trunk control and reactive balance after traumatic injury. In the elderly population, the Hunova has the potential to effectively predict fall risk [34]. In patients after traumatic injuries, the Hunova can effectively restore trunk control and reactive balance [40,41,42].

According to our observations and included studies, researchers have studied platform-based robotic rehabilitation in different phases (acute phase, subacute phase, and chronic phase) and at different time points in patients after injuries. Saglia et al. [11], have summarized the rehabilitation protocol for ankle injuries. In the early phase of ankle therapy, the patient can hardly move his foot. Therefore, passive exercises are usually required, during which the movement parameters such as speed, amplitude, and number of repetitions can be determined by the physical therapist. Active exercises can then help the patient regain ROM to move the ankle fully again. Strength training includes both isometric and isotonic exercises. In the final phase of rehabilitation, the patient must perform proprioceptive training (e.g., balance exercises) [11]. Therefore, we believe that an early-stage intervention leads to a faster recovery of the patient than a late-stage intervention. The reason for this is that patients need to do passive exercises in the initial phase. After that, patients need to do active exercises to regain ROM and proprioceptive training (like balance exercises). In contrast, late-stage patients need more exercise sessions to rehabilitate and recover. However, it would be interesting to investigate this in the future through further studies with larger samples.

Bessler et al. reported about 17 adverse events, including tissue-related, musculoskeletal, and physiological adverse events (adverse blood pressure changes) with the use of stationary gait robots (exoskeletons and end-effector) [59,60]. However, for the platform-based robotic devices, we found insufficient literature on adverse events related to long-term use and training. We are not sure and cannot predict what type of adverse events will occur in patients trained with platform-based robots. Future research may provide clues about the adverse effects. This inability to predict adverse events exists because research in the field of robotic rehabilitation is still in its beginning stage.

Robotic rehabilitation was positively evaluated by physiotherapists and occupational therapists. They reported that patients like to use robotic devices for rehabilitation and that they increase accessibility, autonomy, and comfort, and reduce costs [61,62,63,64,65,66]. Therefore, we believe that physiotherapists will have no problems in using the platform-based robotic devices. However, training is required and the amount of training that physiotherapists need depends on how quickly they grasp and understand the functions of the robotic devices. Indeed, the platform-based robots are user-friendly and widely accepted by physiotherapists [12]. Our study has limitations too. Next to fully peer-reviewed articles, we included in our review gray literature, unpublished data, conference papers, and abstracts, and most of the studied literature presented findings with a low level of evidence (mainly from case studies). Therefore, our results were mainly drawn from studies with a low level of evidence, and we think that to some extent, our conclusions should be considered with caution. Most of the included studies presented significant improvements in ankle performance or gait functionality after performing robotic rehabilitation, although they failed in describing how this was achieved. Also, it was difficult to generalize our findings because several platform-based robotic rehabilitation devices have been tested on individuals with different musculoskeletal or neurological injuries, and it was not possible to adopt universal evaluation criteria with effective outcome measures to determine which was the most effective rehabilitation device [67]. Finally, we limited our search strategy to literature in the English and Italian languages; therefore, some studies may have been excluded, leading to a potentially incomplete search. Lastly, we did not analyze the control strategies, safety, and reliability of the investigated platform-based rehabilitation robots, although this was beyond the scope of the study. This could be a further limitation.

## 5. Conclusions

Our study showed that rehabilitation by using platform-based robots had some encouraging results. The use of robotic rehabilitation allows efficient planning of the rehabilitation process in terms of the duration of sessions, required tools, and availability of a therapist. Therefore, compared to traditional rehabilitation that require combined and intensive efforts of therapists and patients, robotic-assisted rehabilitation should reduce costs because of a shorter hospital stay and greater autonomy at discharge. This highlights the importance of novel rehabilitation techniques that allow therapists to deliver effective treatment interventions while reducing the burden on staff and resources, and related costs. Robotic technology has the potential to transform rehabilitation clinics from labor-intensive to technology-enabled workflows, providing a rich stream of data to help diagnose patients, adjust therapy, and maintain patient records (in a clinic and at home).

Based on our findings, we believe that further studies able to provide results with a higher level of evidence are needed to confirm the effectiveness of platform-based robotic rehabilitation devices. This should primarily include the execution of large sample-size RCTs. These studies should be based on rigorous comparison among the available devices (interventions) and usual care (that is, non-robotic conventional therapies (control)), and should necessarily adopt standardized outcomes to better compare the different models of platform-based rehabilitation robots and to effectively generalize the eventual findings. Therefore, new outcome research is also needed to define universally accepted evaluation criteria able to standardize the devices’ outcomes evaluation. To this end, we also think that wider outcomes should be evaluated, including assessment of comfort, safety, and training performance for the end user.

Despite most studies’ claims that platform-based robots would increase rehabilitation intensity, they allow complex and specialized spatial movements, and the architectures give the robotic device high stiffness, balanced force distribution, and better adaptability to the mechanical properties of human ankles. We believe that this robotic technology can be effectively used by the physiotherapists in the rehabilitation units.

Moreover, in the era of big data and artificial intelligence (AI), computer models can be developed to understand recovery mechanisms, predict the use of different motor control strategies, and ultimately tailor treatment to the patient. We would also like to emphasize that platform-based robotic rehabilitation can only be effective if it would be considered an added value by the patient. Indeed, it is important to consider the perspective of the end user when developing a particular platform-based robotic device to support a specific dysfunction. Such a synergistic effort will certainly lead to effective treatment.

In addition, future research should also focus on the structured and complete recording and dissemination of adverse events related to platform-based robotic rehabilitation to increase knowledge about risks. With this information, appropriate risk mitigation strategies can and should be developed and implemented in platform-based robotic devices to enhance their safety.

Finally, among the platform-based robots studied, the Hunova robot by Movendo technology is commercialized and already available on the market (https://www.movendo.technology/en/) (accessed on 1 August 2021). Therefore, we assume that it has undergone several safety validations before its market launch. In some studies that investigated the Hunova robot, end users also gave positive feedback on training performance and reported that they felt comfortable using the robot for rehabilitation.

## Figures and Tables

**Table 1:** Characteristics of included studies evaluated the effectiveness of Platform-based Robots.

N	Title	Study	Study Type	Clinical Field	Sample Size and Patient Characteristics	Intervention	Measures	Outcomes and Conclusions
1	Development and validation of a robotic multifactorial fall-risk predictive model: A one-year prospective study in community-dwelling older adults	A. Cella, 2020	Longitudinal prospective	Geriatrics	96 subjects (62 females & 34 males); community-dwelling subjects aged ≥65 years	Evaluation of balance components by using the Hunova robot	Model performance (clinical and robotic parameters): Limit of stability (LOS) test, balance test, five times sit-to-stand (FTSS) test.	A multifactorial fall-risk assessment (that includes clinical and Hunova robotic variables) significantly improves the accuracy of predicting the risk of falling in community-dwelling older people.
2	Robotic balance assessment in community-dwelling older people with different grades of impairment of physical performance	A. Cella, 2019	Longitudinal prospective	Geriatrics	96 subjects	Balance parameters assessed by using the Hunova robot in static and dynamic (unstable and perturbating) conditions, in both standing and seated positions and with the eyes open/closed	Balance parameters (sway area, sway path, anterior–posterior and mediolateral range of oscillation of the CoP and the trunk, trunk variability	The multidimensional balance parameters (as detected by the Hunova robotic system) were significantly correlated with SPPB functional performances in community-dwelling older subjects.
3	Effect of a robotic training focused on balance and core stability in Parkinson’s disease: a pilot study	F. Vallone, 2019	Study in clinical practice	Neurology	10 patients with Parkinson’s disease	Traditional and robotic rehabilitation and exercises with Hunova (20 sessions, 2/week)	Balance; limits of stability; mobility (ankle and pelvis) and ankle force; lower limbs strengthening; trunk control; core strengthening	Improvement in the TUG test, to greater pelvis mobility and stability with an improvement in managing the load in sitting position, besides the maintenance of the improvements achieved with traditional treatments on balance, walking speed, stability limits and trunk mobility
4	Effectiveness of robotic balance training on postural instability in patients with mild Parkinson’s disease: A pilot, single-blinded, randomized controlled trial	S. Spina, 2021	A pilot, single-blinded, randomized controlled trial	Neurology	22 subjects	Robotic balance training with Hunova; 20 treatments (45 min/session, 5 times/week)	Primary outcome measures: Mini BEST test (balance impairments), and Berg Balance Scale (static and dynamic balance impairments); secondary outcome measures: 10-meter walk test, FTSS, and Parkinson’s disease questionnaire	Robotic balance training mat have the same effect as conventional balance training on postural stability. Moreover, robotic training could have additional effects in retaining benefit, reducing risk of falls, and improving QoL.
5	Treatment of advanced stage Parkinson disease with hunova: a case study	L. Pendolino, 2019	Single case	Neurology	One patient (70-year-old female) with Parkinson’s disease (IV Hoen and Yahr score)	Ten 1-h sessions with Hunova robot	Sitting postural control; balance in sitting position in static and dynamic conditions; trunk range of motion and control; pelvis range of motion and control; reaching tasks; limits of stability.	Dynamic balance and gait speed functional areas improved; the gait speed increased after the treatment (50% of improvement).
6	Robot-based assessment of sitting and standing balance: preliminary results in Parkinson’s disease	G. Marchesi, 2019	Study in clinical practice	Neurology	Ten people with idiopathic PD (72 + 7 std years old; 2 females) and ten age matched healthy subjects (age: 69 + 8; 4 females) without any history of neurological disease	Performance evaluation by using the Hunova robot (while maintaining upright posture in unperturbed, perturbed, and unstable conditions)	Center of pressure displacement; trunk acceleration with a sensor placed on the sternum	Differences in postural control; bigger trunk oscillations coupled with a sharper increase of the CoP backward displacement was seen at the end of the study.
7	Dynamic Stability and Trunk Control Improvements Following Robotic Balance and Core Stability Training in Chronic Stroke Survivors: A Pilot Study	A. De Luca, 2020	Open randomized controlled clinical trial	Neurology	30 patients; The experimental group (N = 15, mean age 58.53 ± 1.87 SE years, 9 females). The control group (N = 15, mean age 63.46 ± 2.51 SE years, 5 females)	Rehabilitative protocol performed with Hunova robot	Balance performance	Experimental group showed greater improvements in proprioceptive control, reactive balance, and postural control in unstable conditions, compared to the control group, showing an improved trunk control with reduced compensatory strategies at the end of the training.
8	A robot-based assessment of trunk control in Spinal Cord Injured athletes	G. Marchesi, 2020	Study in clinical practice	Neurology	10 subjects (8 healthy subjects and 2 expert sit-ski athletes affected by paraplegia after SCI)	Proposed exercises in the protocol performed with Hunova robot	Trunk control	Differences in motor performance was observed at the end of the study
9	The use of the robotic device hunova as rehabilitation and evaluation tool for functional balance in individuals with spinal cord injury	A. Leo, 2018	Study in clinical practice	Neurology	8 subjects (5 M 3 F, mean time from disease 12 ± 5.74, mean age 46 ± 10.6 years) in chronic condition	Evaluation and training sessions performed with Hunova robot	Balance, trunk control, dual motor task with movements of the upper limbs, strengthening, core stability	The subjects showed improvements in trunk control measured both by clinical scales and by Hunova robot during active control tasks and balance tasks in seated position.
10	The role of hunova in rehabilitative treatment of functional balance in a patient with complete spinal cord injury (SCI)	A. Leo, 2019	Single case	Neurology	One patient (46-year-old male) with complete neurological loss	20 sessions (2 sessions per week, 10 weeks) with Hunova	Balance; trunk control; dual-motor-task with movements of the upper limbs; strengthening; core stability	The score of the sitting balance assessment for spinal cord injury (SBASCI) scale improved (from 29 to 37.5). Improved trunk control both in balancing and perturbating conditions and performing active movements with trunk.
11	Balance assessment and training in an elderly neurological patient: a case study	M. Escelsior, 2020	Single case	Neurology	One patient (60-year-old male); retro-ocular frontal aneurysm with hemorrahage	Eleven 1-h sessions, twice a week with Hunova	Trunk compensations; trunk oscillations; balance; functional lower extremity strength	Dynamic balance and gait speed functional areas improved at the end of the training program; the gait speed increases after the treatment (50% of improvement).
12	The role of Hunova in the treatment of patient with a cavernous malformation and hypertrophic olivary degeneration	U. Nguyen, 2021	Single case	Neurology	One patient; 59-year-old male, former professional hockey player (with a cavernous malformation and hypertrophic olivary degeneration)	7 customized Hunova training	Monopodalic equilibrium, bipodalic equilibrium	Clinical scales showed improved gait pattern, balance, gait endurance. The improvements obtained in the robotic tests showed increased balance in static and dynamic situations also.
13	Rehabilitation of somatoparaphrenia with misoplegia: insights from a single case-pilot study	M.G. Maggio, 2021	Single case	Neurology	One case	Novel multidisciplinary treatment (motor rehabilitation training, traditional physiotherapy and robotic rehabilitation using the Hunova robot and psychological counselling)	Neuropsychological evaluation focused on body representation (BR), cognitive and linguistic functions	The integrated psychological and motor approach may effectively rehabilitate patients with SP, even in the presence of misoplegia.
14	Use of hunova for rehabilitation following severe acquired brain injury (ABI): a case study	M. Burlando, 2019	Single case	Neurology	One patient (20-year-old woman) with chronic ABI	10 sessions (2 sessions per week, 5 weeks) with Hunova.	Bipodalic equilibrium; monopodalic equilibrium; ankle range of motion; balance; core stability	Subject improved her performance in terms of gait speed (20%) and ankle range of motion (46%) assessed by physiotherapists and control of balance in robotic tests.
15	Proprioceptive and motor training using the high-performance robotic device hunova: preliminary results of a randomized, controlled trial in patients with lower limb post-traumatic conditions	E. Taglione, 2018	Open randomized controlled clinical trial	Orthopedics	44 subjects (mean age 45.34 ± 10.41 years): 22 subjects (19 M, 3 F, mean age 45.86 ± 10.93, 10 with proximal and 12 with distal injury) were in experimental group, 22 subjects (16 M, 6 F, mean age 44.81 ± 10.09, 9 with proximal and 13 with distal injury)	A rehabilitation training with Hunova (for 3 weeks) robot.	Balance; limits of stability; endurance proprioceptive; lower extremity	The performance of the experimental group in proprioceptive tests improved significantly compared to the control group (decrease in figural errors in the drawing task, *p* = 0.03; increase in the number of targets in the grasping test, *p* = 0.02). Hunova allows one to measure significant parameters of static and dynamic stability and can centralize a complex progression of exercises to recover trunk control and reactive balance after traumatic injuries. Training with Hunova robot was as effective as traditional treatment.
16	Evaluation and rehabilitation training with hunova robotic system for the recovery of dynamic postural stability: open randomized interventional protocol, on patients after ACL surgical reconstruction	F. Vallone, 2018	Open randomized controlled clinical trial	Orthopedics	10 subjects	A rehabilitation training with Hunova (for 8 weeks) robot	Reinforcement; balancing; proprioceptive and core stability	Hunova robot has proven to be safe, easy to use, as effective as conventional treatment, and highly efficient, supporting with its functions all programs of joint mobilization, muscle work, and proprioceptive recovery that were used in the control group.
17	Hunova as a tool in rehabilitation following robotic knee arthroplasty	A. Di Matteo, 2019	Case study	Orthopedics	24 subjects (12 subjects in control group and 12 subjects in experimental group)	8 sessions (4 sessions per week, for 2 weeks) with Hunova	Weight-bearing sensibilization; proprioception and control; balance; lower-limb strength; core stability and postural passages; BI, KRS, NPRS	Experimental group has better results, compared to control group, in orthostatic stability, postural passages such as sit to stand and in reactive balance at 3-month follow-up.
18	Rehabilitative treatment of fracture-dislocation of the ankle with hunova: a case study	V. Da Pieve, 2019	Single case	Orthopedics	One patient (48 years old female) admitted to rehabilitation facility 1 month after surgery for an ankle fracture	8 sessions (30 min sessions, 2 sessions per week, 1 month) with Hunova	Ankle range of motion; torque; trunk compensations	The left ankle performance improves in terms of ROM and isometric and isokinetic force, and it reaches a good symmetry with the right ankle (<10%). Consequently, balance control increases.
19	Hunova for performance optimization of the sccocer player: a case study	P. Barbero, 2020	Single case	Orthopedics	One patient (male, 19 years old, professional youth league U-19 soccer player)	Ten 1-h weekly sessions with Hunova.	Core; ankle range of motion; ankle force; monopodalic equilibrium; bipodalic equilibrium and squat.	The overall performance index score improved from 61% to 76%. The two trained functional areas (core and ROM) reached the average range of performance of the entire team.
20	Hunova for evaluation and treatment of chronic ankle instability using performance index	G. Risicato, 2020	Single case	Orthopedics	One patient (24 years old professional basketball player affected by chronic ankle instability)	Eight 1-h sessions, twice a week with Hunova	Ankle range of motion; monopodalic equilibrium; bipodalic equilibrium and squat	The overall performance index score improved from 57% to 74%. The two trained functional areas (core and ROM) reached the average range of performance. In addition, the FAAM score was improved.
21	Ankle rehabilitation using the high-performance robotic device ARBOT: results from a randomised controlled trial	E. Taglione, 2015	Randomized controlled clinical trial	Orthopaedics	32 subjects (31 completed the study)	A rehabilitation training with ARBOT robot	Dorsiflexion ROM; isometric and isokinetic plantar-flexion torque; proprioceptive performance	ARBOT demonstrated to be safe, reliable, and easy to manage. The study showed that the training with ARBOT was totally comparable to traditional methods.
22	Orthopedic Rehabilitation Using the “Rutgers Ankle” Interface	M. Girone, 2000	Case study	Orthopedics	4 subjects (2 patients exhibited hypermobility secondary to chronic ankle instability and the other 2 presented with hypomobility as the sequelae of fractures)	Rutgers ankle prototype	Displacement and torque	The displacement of the uninvolved leg was comparable to normal ROM at the ankle; the maximum torque generated by the uninvolved limb was much larger than that generated by the involved limb.
23	Rehabilitation of Musculoskeletal Injuries Using the Rutgers Ankle Haptic Interface: Three Case Reports	J. E. Deutsch, 2001	Single-case series	Orthopedics	3 subjects; musculoskeletal ankle injuries	Rutgers ankle system with a 3-D piloting of an airplane	ROM; torque generation capacity and ankle mechanical work	Task accuracy improved to 100% for Case 1; a fivefold increase in ankle power output for Case 2 and a three-fold increase for Case 3; both Case 2 and Case 3 reached 100% task accuracy.
24	Post-stroke rehabilitation with the rutgers ankle system: a case study	J. E. Deutsch, 2001	Before–after, single case	Neurology	1 subject; a left cerebral vascular accident	Rutgers ankle system with a 3-D piloting of an airplane	Ankle and foot mobility; force generation; coordination; ability to walk and climb stairs	Strength; endurance; task accuracy; coordination; walking and stair-climbing ability improved over six rehabilitation sessions
25	Virtual reality-based system for ankle rehabilitation post stroke	R. F. Boian, 2002	Single case series	Neurology	3 subjects with post-stroke	Rutgers ankle with two video games	Power and walking endurance	Increase in power generation for all motions and walking endurance increase for one patient
26	Haptic effects for virtual reality-based post-stroke rehabilitation	R. F. Boian, 2003	Single case series	Orthopedics	3 subjects; 2 patients had normal sensation and the third had a decrease with 8/12 on the FM lower extremity sensory score	Second version of VR based ankle rehabilitation system	Muscle strength	Subject 1 increased strength in all four muscle groups, subject 2 in two muscle groups and subject 3 in three muscle groups
27	Improved gait and elevation speed of individuals post-stroke after lower extremity training in virtual environments	J. E. Deutsch, 2004	Single case series	Neurology	6 post-stroke subjects	A robotic device (Rutgers ankle was the input to the virtual environment)	Gait and elevation speed	Gait speed increased 11% (*p* = 0.08) and elevation time decreased 14% (*p* = 0.05); gait endurance increased 11%; gait and elevation speed improved from 0 to 44% and 3 to 33%
28	Ankle control and strength training for children with cerebral palsy using the Rutgers ankle CP: a case study	D. Cioi, 2011	Single case	Neurology	1 subject (a child with mild ataxic CP)	Rutgers ankle CP	Impairment, function, and quality of life	Strength, motor control, gait function, overall function, and quality of life improved.
29	Robotics and Gaming to Improve Ankle Strength, Motor Control, and Function in Children with Cerebral Palsy—A Case Study Series	G. C. Burdea, 2012	Case study	Neurology	3 subjects; male children with CP	Rutgers ankle CP	Impairment, function, quality of life and game performance	Strength, motor control, gait function, overall function, quality of life, and game performance improved.
30	Technical and patient performance using a virtual reality-integrated telerehabilitation system: preliminary finding	J.E. Deutsch, 2007	Before–after	Neurology	6 post-stroke subjects	Rutgers ankle prototype robot with VR	Accuracy of ankle movement; exercise duration; training efficiency; mechanical power of ankle; number of repetitions	All measures improved in the first three weeks and did not decrease during the transition.
31	Effects of Training with a Robot-Virtual Reality System Compared with a Robot Alone on the Gait of Individuals After Stroke	A. Mirelman, 2008	Randomized controlled trial	Neurology	18 chronic hemiparesis after stroke patients	Rutgers Ankle Rehabilitation System coupled with VR	Velocity and distance walked	Greater changes in velocity and distance walked were demonstrated for the group trained with the robotic device coupled with the VR than training with the robot alone.
32	Intelligent Stretching of Ankle Joints with Contracture/Spasticity	L. Q. Zhang, 2002	Before–after, Case control	Neurology	9 subjects (5 healthy subjects and 4 chronic stroke patients with ankle contracture and/or spasticity)	A custom-designed joint-stretching device	ROM; joint stiffness; viscous damping; reflex excitability	The passive and active ROM of the ankle joint increased; joint stiffness and viscosity were reduced; reductions in reflex excitability
33	Feedback-Controlled and Programmed Stretching of the Ankle Plantarflexors and Dorsiflexors in Stroke: Effects of a 4-Week Intervention Program	R. W. Selles, 2005	Single-case series	Neurology	10 spasticity and/or contracture after stroke subjects	Feedback controlled and programmed stretching device	ROM; muscle strength; joint stiffness; joint viscous damping; reflex excitability; walking speed; subjective experiences	Significant improvements were found in the passive ROM, maximum voluntary contraction, ankle stiffness, and comfortable walking speed.
34	Combined passive stretching and active movement rehabilitation of lower-limb impairments in children with cerebral palsy using a portable robot	Y-N. Wu, 2011	Before–after	Neurology	12 children with CP	A portable rehabilitation robot with computer game	PROM, AROM, dorsiflexor and plantar flexor muscle strength; selective control assessment of the lower extremity and functional outcome measures	Improvements in dorsiflexion PROM (*p* = 0.002), AROM (*p* = 0.02), and dorsiflexor muscle strength (*p* = 0.001); spasticity of the ankle musculature was reduced (*p* = 0.01); selective motor control improved (*p* = 0.005); functionally, participants improved balance (*p* = 0.0025) and increased walking distance within 6 min (*p* = 0.025)
35	Effects of robot-guided passive stretching and active movement training of ankle and mobility impairments in stroke	G. Waldman, 2013	Before–after	Neurology	24 stroke survivors with impaired ankle motor function	18 sessions (3 times a week over 6 weeks) with portable ankle rehabilitation robot	Active dorsiflexion range; dorsiflexor muscle strength; the average MAS, STREAM, and Berg balance	Dorsiflexion active range motion and dorsiflexor muscle strength, MAS, STREAM, and Berg balance significantly improved in the intervention group.
36	Development of ankle dorsiflexion/plantarflexion exercise device with passive mechanical joint	K. Homma, 2007	Case control, Single case	Neurology	5 subjects (4 healthy subjects and a male with hemiplegia)	A passive exercise device for ankle dorsiflexion and plantarflexion	ROM and pressure distribution	Improvements were within the margin of the measuring error.
37	Assisted movement with enhanced sensation (AMES): coupling motor and sensory to remediate motor deficits in chronic stroke patients	P. Cordo, 2009	Before-After	Neurology	11 post-stroke and severe motor disability of the lower extremity patients	AMES treatment device for ankles	Strength; joint position; motor function	Strength increased 10% in most ankles; joint position improved 10% in all ankles; motor function improved significantly.
38	A proprioceptive neuromuscular facilitation integrated robotic ankle–foot system for poststroke rehabilitation	Z. Zhou, 2015	Case-control	Neurology	10 subjects (five normal subjects-age 27.2 ± 1.8 years; five post stroke patients-age 65.6 ± 9.0 years)	PNF treatment with integrated robotic ankle–foot rehabilitation system carried out for 6 weeks (3 times a week)	Passive and active joint properties	PNF integrated robotic ankle–foot rehabilitation system is effective in improving ankle spasticity and/or contracture.

## Data Availability

The data presented in this study are available on request from the corresponding author.

## Supplementary Materials

The following supporting information can be downloaded at: https://www.mdpi.com/article/10.3390/bioengineering9040129/s1, Table S1: Summary of quality assessments using JBI appraisal checklist; Table S2: Summary of quality assessments (Longitudinal Prospective and RCTs) using JBI appraisal checklist.
