# Supplementary material for: Effectiveness of Platform-Based Robot-Assisted Rehabilitation for Musculoskeletal or Neurologic Injuries: A Systematic Review
Source: Bioengineering (Basel). 2022 Mar 22;9(4):129. doi: 10.3390/bioengineering9040129 (PMC9029074; doi:10.3390/bioengineering9040129)
Supplement: Supplementary file 1 [file bioengineering-09-00129-s001.zip › bioengineering-1634682-supplementary.pdf]

Table S1. Summary of quality assessments using JBI appraisal checklist.

Author	Study type	Items on Joanna Briggs Institute								Raw score and %	Risk
		Q1	Q2	Q3	Q4	Q5	Q6	Q7	Q8		
F.Vallone (2019)	Clinical practice	0	0	1	1	1	1	1	1	6/8 = 75.0%	Low
G.Marchesi (2019)	Clinical practice	1	1	1	1	1	1	1	1	8/8 = 100%	Low
G.Marchesi (2020)	Clinical practice	0	1	1	1	1	1	1	1	7/8 = 87.5%	Low
A.Leo (2018)	Clinical practice	0	0	1	1	1	U	0	1	4/8 = 50.0%	Moderate
M.G.Maggio (2021)	Single case	1	1	1	1	1	U	1	1	7/8 = 87.5%	Low
M. Girone (2000)	Case study	0	0	1	1	1	1	1	1	6/8 = 75.0%	Low
J. E. Deutsch (2001)	Case series	0	0	1	1	1	1	1	1	6/8 = 75.0%	Low
J. E. Deutsch (2001)	Before-after, Single case	1	1	1	U	1	1	U	1	6/8 = 75.0%	Low
R. F. Boian (2002)	Case series	0	1	1	1	1	1	1	1	7/8 = 87.5%	Low
R. F. Boian (2003)	Case series	0	0	1	1	1	U	1	1	5/8 = 62.5%	Moderate
J. E. Deutsch (2004)	Case series	0	1	1	1	1	U	1	1	6/8 = 75.0%	Low
R. W. Selles (2005)	Single case series	1	1	1	1	1	1	1	1	8/8 = 100%	Low
D. Cioi (2011)	Single case	0	0	1	1	1	1	1	1	6/8 = 75.0%	Low
G. C. Burdea (2012)	Case study	1	1	1	1	1	1	1	1	8/8 = 100%	Low
L.Q.Zhang (2002)	Before-after, case control	0	1	1	1	1	1	U	1	6/8 = 75.0%	Low
J.E. Deutsch (2007)	Before-after	0	0	1	1	1	U	U	1	4/8 = 50.0%	Moderate
K. Homma (2007)	Case control, single case	0	1	1	1	0	u	1	1	5/8 = 62.5%	Moderate
P. Cordo (2009)	Before-after	0	1	1	1	1	1	1	1	7/8 = 87.5%	Low
Y-N. Wu (2011)	Before-after	1	1	1	1	1	1	1	1	8/8 = 100%	Low
G. Waldman (2013)	Before-after	0	1	1	1	1	1	U	1	6/8 = 75.0%	Low
L. Pendolino (2019)	Single case	0	1	1	1	U	1	0	1	5/8 = 62.5%	Moderate
M. Burlando (2019)	Single case	0	1	1	1	1	1	1	1	7/8 = 87.5%	Low
A. Leo (2019)	Single case	0	1	1	1	0	1	0	1	5/8 = 62.5%	Moderate
A. Di Matteo (2019)	Case study	0	1	1	1	1	1	0	1	6/8 = 75.0%	Low
V. Da Pieve (2019)	Single case	0	1	1	1	1	1	1	1	7/8 = 87.5%	Low
M. Escelsior (2020)	Single case	0	1	1	1	1	U	0	1	5/8 = 62.5%	Moderate
P. Barbero (2020)	Single case	0	1	1	1	1	U	0	1	5/8 = 62.5%	Moderate
G. Risicato (2020)	Single case	1	1	1	U	1	0	0	1	5/8 = 62.5%	Moderate
U.Nguyen (2021)	Single case	0	1	1	1	1	1	1	1	7/8 = 87.5%	Low
Z. Zhou (2015)	Case-control	1	1	1	1	1	0	1	1	7/8 = 87.5%	Low

JBI Criteria to be scored: Q1. Were patient's demographic characteristics clearly described; Q2. Was the patient's history clearly described and presented as a timeline; Q3. Was the current clinical condition of the patient on presentation clearly described; Q4. Were diagnostic tests or assessment methods and the results clearly described; Q5. Was the intervention(s) or treatment procedure(s) clearly described; Q6. Was the post-intervention clinical condition clearly described; Q7. Were adverse events (harms) or unanticipated events identified and described; Q8. Does the case report provide takeaway lessons. 1 = Yes, 0 = No, and U = Unclear.

Abbreviations: 1 = Yes; 0 = No; U = Unclear; NA = Not Applicable; JBI: Joanna Briggs Institute

Criteria used to rank the risk of bias

i) ≤49% = high risk of Bias

ii) 50% and 69% = Moderate risk of Bias (9 studies)

iii) Above 70% = low risk of Bias (21 studies)

Table S2. Summary of quality assessments (Longitudinal Prospective and RCTs) using JBI appraisal checklist.

Author	Study type	Items on Joanna Briggs Institute tool													Raw score and %	Risk
		Q1	Q2	Q3	Q4	Q5	Q6	Q7	Q8	Q9	Q10	Q11	Q12	Q13		
A.De Luca (2020)	RCT	1	0	1	1	0	1	0	1	1	1	1	1	1	10/13 = 77.0%	Low
E.Taglione (2015)	RCT	1	0	0	1	0	U	0	1	U	0	1	U	1	5/13 = 38.5%	High
E.Taglione (2018)	RCT	1	0	0	0	0	U	1	U	1	0	1	U	U	4/13 = 30.8%	High
F.Vallone (2018)	RCT	1	0	0	0	0	0	U	1	U	1	1	1	1	6/13 = 46.0%	High
S.Spina (2021)	RCT	1	0	0	1	1	1	U	1	1	1	1	U	1	9/13 = 69.2%	Moderate
A.Mirelman (2008)	RCT	1	1	0	1	1	U	0	1	1	1	1	1	1	10/13 = 77.0%	Low
A. Cella (2020)	Longitudinal prospective	0	1	1	1	1	1	1	0	1	0	1	NA	NA	8/11 = 72.7%	Low
A. Cella (2019)	Longitudinal prospective	0	1	1	1	1	1	1	0	1	0	1	NA	NA	8/11 = 72.7%	Low

JBI Critical Appraisal Checklist for RCTs to be scored: Q1. Was true randomization used for assignment of participants to treatment groups; Q2. Was allocation to treatment groups concealed; Q3. Were treatment groups similar at the baseline; Q4. Were participants blind to treatment assignment; Q5. Were those delivering treatment blind to treatment assignment; Q6. Were outcomes assessors blind to treatment assignment; Q7. Were treatment groups treated identically other than the intervention of interest; Q8. Was follow up complete and if not, were differences between groups in terms of their follow up adequately described and analyzed; Q9. Were participants analyzed in the groups to which they were randomized; Q10. Were outcomes measured in the same way for treatment groups; Q11. Were outcomes measured in a reliable way; Q12. Was appropriate statistical analysis used; Q13. Was the trial design appropriate, and any deviations from the standard RCT design (individual randomization, parallel groups) accounted for in the conduct and analysis of the trial.

JBI Critical Appraisal Checklist for Longitudinal prospective Studies to be scored: Q1. Were the two groups similar and recruited from the same population; Q2. Were the exposures measured similarly to assign people to both exposed and unexposed groups; Q3. Was the exposure measured in a valid and reliable way; Q4. Were confounding factors identified; Q5. Were strategies to deal with confounding factors stated; Q6. Were the groups/participants free of the outcome at the start of the study (or at the moment of exposure); Q7. Were the outcomes measured in a valid and reliable way; Q8. Was the follow up time reported and sufficient to be long enough for outcomes to occur; Q9. Was follow up complete, and if not, were the reasons to loss to follow up described and explored; Q10. Were strategies to address incomplete follow up utilized; Q11. Was appropriate statistical analysis used?

Abbreviations: 1 = Yes; 0 = No; U = Unclear; RCT = Randomized controlled trial; NA = Not Applicable; JBI: Joanna Briggs Institute

Criteria used to rank the risk of bias:

i) ≤49% = high risk of Bias (3 studies)

ii) 50% and 69% = Moderate risk of Bias (1 study)

iii) above 70% = low risk of Bias (4 studies)
